# Template-Directed Ligation of Tethered Mononucleotides by T4 DNA Ligase for Kinase Ribozyme Selection

**DOI:** 10.1371/journal.pone.0012368

**Published:** 2010-08-24

**Authors:** David G. Nickens, Nirmala Bardiya, James T. Patterson, Donald H. Burke

**Affiliations:** 1 Department of Chemistry, Indiana University, Bloomington, Indiana, United States of America; 2 Department of Biology, Indiana University, Bloomington, Indiana, United States of America; 3 Department of Molecular Microbiology and Immunology and Department of Biochemistry, University of Missouri, Columbia, Missouri, United States of America; University of Helsinki, Finland

## Abstract

**Background:**

*In vitro* selection of kinase ribozymes for small molecule metabolites, such as free nucleosides, will require partition systems that discriminate active from inactive RNA species. While nucleic acid catalysis of phosphoryl transfer is well established for phosphorylation of 5′ or 2′ OH of oligonucleotide substrates, phosphorylation of diffusible small molecules has not been demonstrated.

**Methodology/Principal Findings:**

This study demonstrates the ability of T4 DNA ligase to capture RNA strands in which a tethered monodeoxynucleoside has acquired a 5′ phosphate. The ligation reaction therefore mimics the partition step of a selection for nucleoside kinase (deoxy)ribozymes. Ligation with tethered substrates was considerably slower than with nicked, fully duplex DNA, even though the deoxynucleotides at the ligation junction were Watson-Crick base paired in the tethered substrate. Ligation increased markedly when the bridging template strand contained unpaired spacer nucleotides across from the flexible tether, according to the trends: A_2_>A_1_>A_3_>A_4_>A_0_>A_6_>A_8_>A_10_ and T_2_>T_3_>T_4_>T_6_≈T_1_>T_8_>T_10_. Bridging T's generally gave higher yield of ligated product than bridging A's. ATP concentrations above 33 µM accumulated adenylated intermediate and decreased yields of the gap-sealed product, likely due to re-adenylation of dissociated enzyme. Under optimized conditions, T4 DNA ligase efficiently (>90%) joined a correctly paired, or T∶G wobble-paired, substrate on the 3′ side of the ligation junction while discriminating approximately 100-fold against most mispaired substrates. Tethered dC and dG gave the highest ligation rates and yields, followed by tethered deoxyinosine (dI) and dT, with the slowest reactions for tethered dA. The same kinetic trends were observed in ligase-mediated capture in complex reaction mixtures with multiple substrates. The “universal” analog 5-nitroindole (dNI) did not support ligation when used as the tethered nucleotide.

**Conclusions/Significance:**

Our results reveal a novel activity for T4 DNA ligase (template-directed ligation of a tethered mononucleotide) and establish this partition scheme as being suitable for the selection of ribozymes that phosphorylate mononucleoside substrates.

## Introduction

Artificial ribozymes can be selected *in vitro* to catalyze diverse chemical reactions [1], [2], [3], [4]. As such, they provide unique opportunities to engineer metabolic pathways, to expand the tool kit of tailor-made devices for synthetic biology, and to test RNA world theories of the earliest evolution of life. Ribozyme selection usually involves either physical sequestration of rare active species or their preferential amplification. One of the current fundamental challenges for expanding the catalytic scope of nucleic acids is to identify RNAs that act upon natural or engineered metabolites. A strategy that has met with some success has been to tether the targeted small-molecule substrate to an RNA library through a flexible linker, then recover RNA species that convert the tethered substrate into tethered product [5]. The tether ensures linkage between the product and the ribozyme that produced it, while also giving the substrate some freedom to explore the surface of the folded RNA. If the linker is relatively inert, substrate retention in the active site is enhanced if the evolving ribozyme provides at least some of the interactions that would be required to bind free (untethered) substrate. A second challenge that applies especially to group transfer and condensation reactions is that selections often yield ribozymes that modify themselves within the RNA chain rather than on the tethered substrate. This is likely due to entropic barriers being lower for organizing the 2′OH than the quasi-diffusible tethered substrate [6], [7]. New approaches and technologies are needed to circumvent these problems. We propose that when the intended substrate is a tethered *mono*nucleoside(tide), an enzymatic partition step can enforce selection of the intended activity at the targeted site by exploiting the activities of enzymes such as DNA ligase that normally act upon *poly*nucleotides. The present work establishes the proof-of-concept for a strategy that enables the selection of nucleoside kinase ribozymes.

Phosphorylation is among the most important group transfer reactions in biology, and it is an especially attractive model reaction for studying nucleic acid catalysis of metabolically meaningful reactions. For RNA world biology, the ability to form nucleotide monophosphates (NMPs) from nucleosides and a suitable phosphoryl donor would have aided the synthesis of activated monomers for nucleic acid synthesis. In the context of synthetic biology, the growth and replication of artificial, cell-like vesicles is stimulated by increasing the internal osmolarity [8], [9]; thus, nucleoside phosphorylation within such vesicles should decrease nucleoside diffusion across the membrane, drive NMP accumulation within their interiors, and stimulate vesicle growth. In medicine, many of the nucleoside analog prodrugs used in anti-cancer and anti-viral therapies must be monophosphorylated upon entering cells to avoid enzymatic degradation. While several groups have described *poly*nucleotide kinase ribozymes that phosphorylate 5′ or internal 2′ OH groups [6], [7], [10]–[18], the successful selection of kinase ribozymes for *mono*nucleoside phosphorylation would be a significant advance for RNA catalysis, help to constrain RNA world ribozymology, generate new tools for synthetic biology and enable new strategies for increasing the therapeutic potency of nucleoside prodrugs.

**Figure 1:**
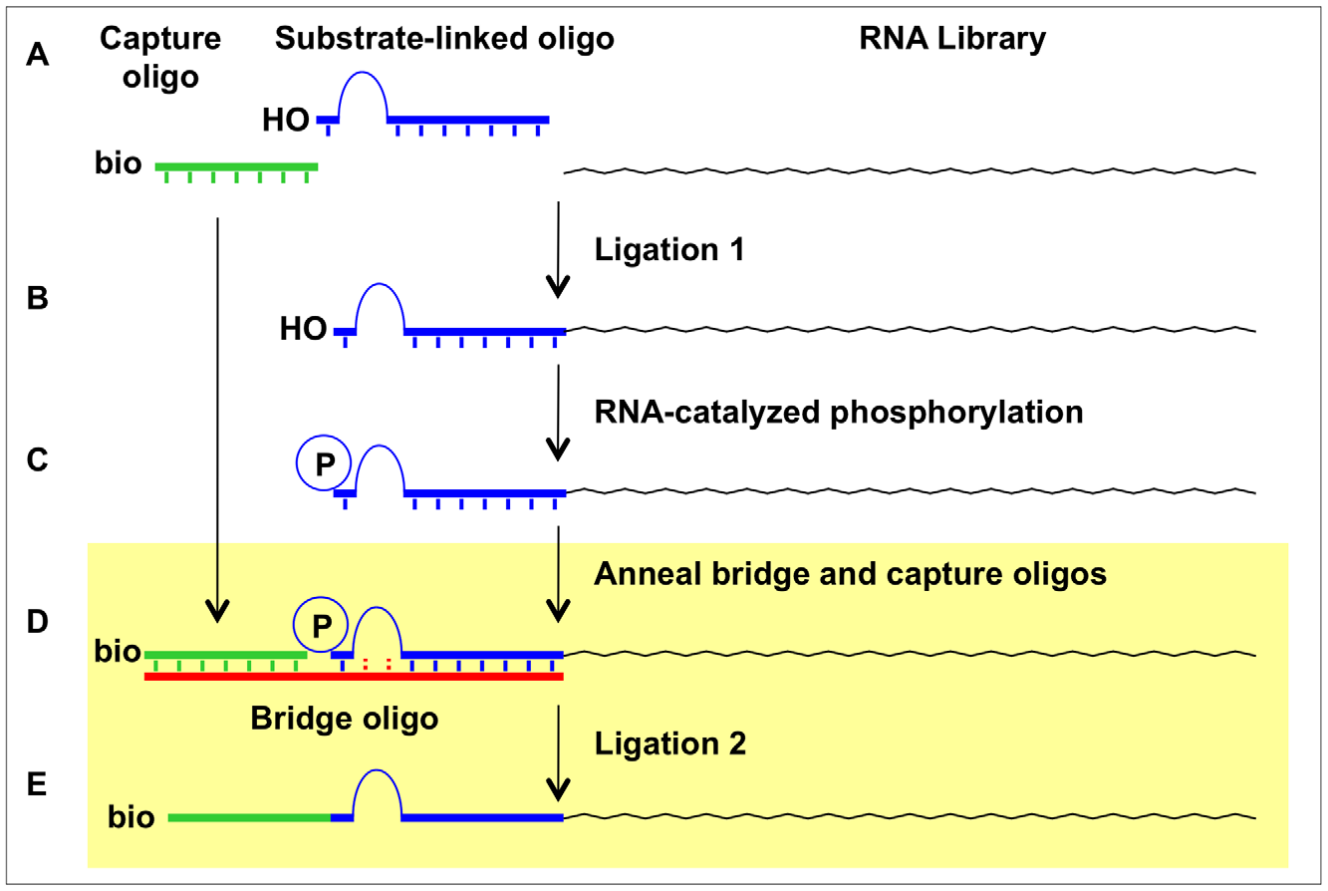
Potential ligation-based selection strategy for identifying nucleoside kinase ribozymes. RNA library is first ligated to a substrate-linked oligonucleotide (**A**) to generate a substrate-linked library (**B**). RNA molecules that phosphorylate the tethered mononucleoside under suitable conditions (**C**) are identified by first annealing them to the bridging and capture oligos (**D**) and joining them via ligation (**E**). These last two steps are the subject of the current study (shaded box).

Kinase ribozymes for polynucleotide substrates have been selected in previous works by incubating random-sequence RNA libraries with ATPγS and/or GTPγS. Species that acquired one or more sulfurs through thio-phosphoryl transfer were then recovered by taking advantage of the unique chemical properties of the sulfur, such as capture on a polyacrylamide gel with an organomercurial layer. When a similar strategy was applied to RNA populations carrying tethered substrates, all of the ribozymes from the final libraries phosphorylated internal 2′ OHs, with a non-random preference for phosphorylation on guanosine 2′ OHs [6], [7]. While self-modifying kinase ribozymes present rich opportunities for understanding the structures and mechanisms of nucleic acid catalysis, it is unlikely that their active sites can be remodeled to accommodate mononucleosides or other metabolites for phosphorylation. A different strategy was used by Li and Breaker to identify self-kinasing DNAzymes: single-stranded DNA molecules catalyzed autophosphorylation at the 5′ position converted themselves into substrates for ligation to an oligonucleotide, which then served as primer binding site for PCR amplification [15]. Because enzymatic ligation by DNA ligases is well known to require a 5′ phosphate, this approach strictly enforced that phosphorylation take place on the 5′ terminal OH of the polynucleotide chain. It should be possible to adapt a ligation-based approach, such as the one outlined in **Fig. 1**, for the selection of mononucleoside kinase ribozymes, provided that DNA ligase can be made to accept a tethered mononucleotide (the phosphorylated product of the RNA-catalyzed kinase reaction) as a substrate for ligation.

**Figure 2:**
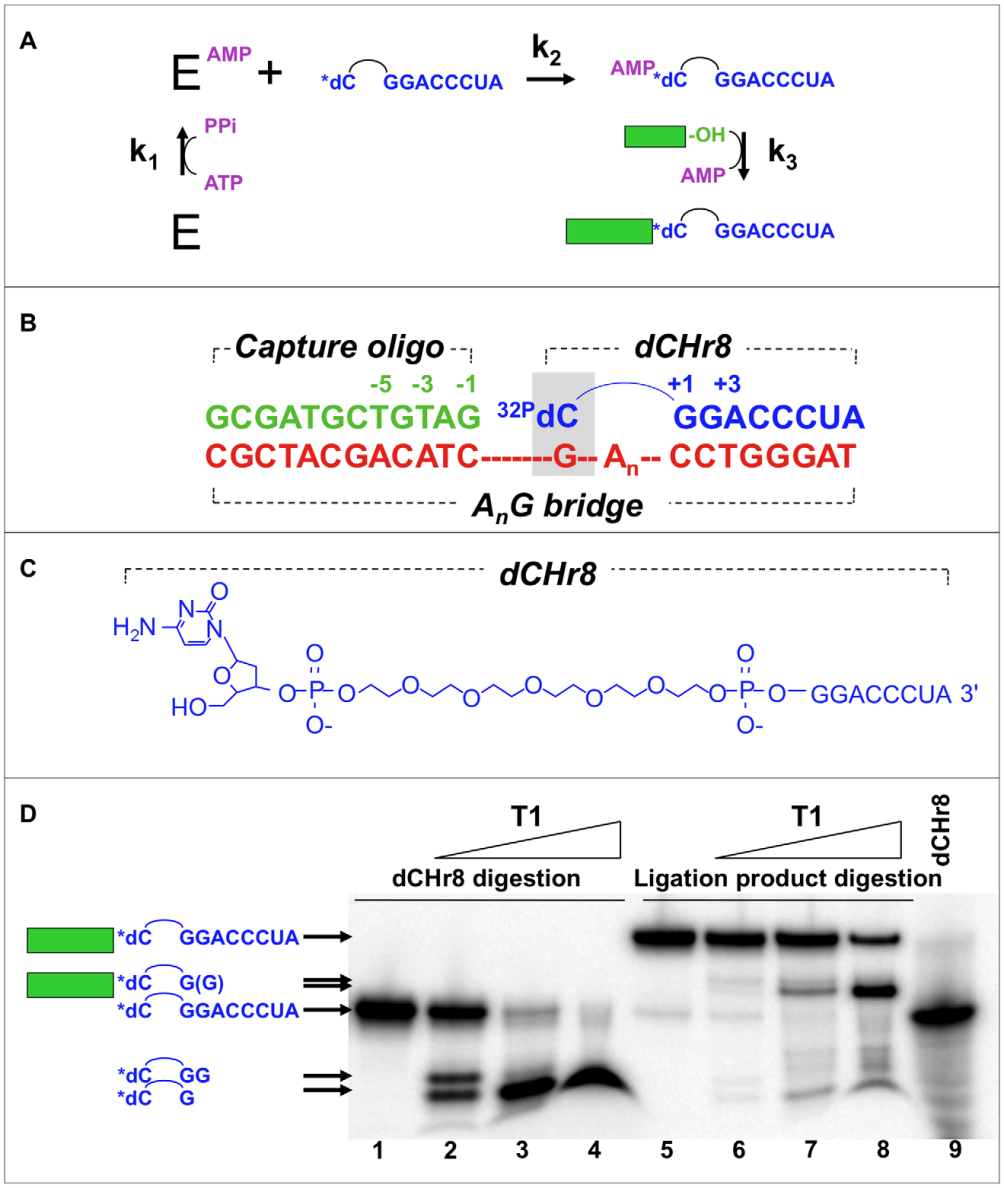
Experimental system for evaluating minimal tethered junctions. **A**) Kinetic scheme for ligation showing the three group transfer reactions in the ligation mechanism, as described in the text (bridging strand omitted for clarity). **B**) Minimal ligation junction including the annealed dCHr8 : capture oligo : A_n_G bridge oligonucleotides, where n is the number of intervening nucleotides. HEG linker within dCHr8 is shown as an arc. Shaded box, dC∶dG base pair at the ligation junction. Analogous junctions using HEG-G, A_n_Y or T_n_Y bridges are described in the text. **C**) The dCHr8 oligoribonucleotide used as the downstream oligo of the ligation complexes. Other downstream oligoribonucleotides, referred to collectively as dXHr8, are identical in structure except for the attached nucleobase. **D**) Identification of ligated product. Ligation product and control unligated dCHr8 were gel purified and digested with T1 ribonuclease at increasing concentrations (wedge above lanes) using 0, 0.01, 0.1, and 1.0 U/µL enzyme. Tethered mononucleotide is radiolabeled (asterisk). Compositions of the major products are shown to the left. Capture oligo strand is represented as a filled rectangle and HEG as an arc; RNA sequences are shown explicitly.

The suitability of a ligation-based approach is governed by the mechanism of ligase enzymes. DNA ligases from viruses, bacteriophage, archaea and eukaryotes couple the strand-joining, or “gap-sealing,” reaction to cleavage of the α−β phosphodiester bond of ATP in a three-step mechanism (**Fig. 2A**) [19], [20]. In the first step (“enzyme charging”), an active site lysine attacks the α phosphate of ATP, displacing pyrophosphate and forming a metastable phosphoramidate linkage. Pyrophosphate addition can reverse this step, while pyrophosphate hydrolysis makes it effectively irreversible. NAD^+^ donates the adenylate in the first step of the corresponding bacterial ligases [20]. In the second step (“adenylate formation”), the 5′ phosphate oxyanion from the downstream fragment in the nicked DNA attacks the phosphoramidate α phosphate to regenerate the active site lysine and to produce a 5′,5′-linked adenylate intermediate. In the third step (“gap-sealing”), the 3′ OH of the upstream fragment attacks the 5′ phosphate of the downstream fragment, displacing AMP and sealing the gap. A tethered mononucleotide produced by a kinase ribozyme during selection is expected to be a suboptimal ligation substrate for DNA ligase. Nevertheless, structural contexts other than standard B-form DNA-DNA duplexes are known to be compatible with enzymatic ligation. For example, two RNA fragments annealed to a bridging oligo*deoxy*nucleotide can be ligated using bacteriophage T4 DNA ligase [21]. Mispaired DNA junctions can be ligated under certain conditions [22], as can junctions that contain nucleotide analogs [23].

The present work examines enzymatic ligation of an upstream “capture oligo” to a downstream monodeoxynucleotide that is tethered to the 5′ end of an 8-mer oligoribonucleotide. The ligation junction is stabilized by annealing both fragments with a complementary bridging oligo (**Fig. 2B**). The 5′ monophosphate on the tethered mononucleotide mimics the product of phosphorylation by a kinase ribozyme, so that the subsequent ligase-catalyzed reaction simulates the partition phase of a selection scheme wherein active ribozymes are preferentially recovered. We find that ligation can be driven to near completion for most tethered mononucleotides under optimal conditions. Mismatches and high concentrations of ATP drive accumulation of adenylated intermediates, with only slow, partial conversion to ligated product, likely limited by enzyme re-adenylation. We measured rates of the individual steps in the ligation reaction (adenylation and gap-sealing) under optimized conditions on a variety of tethered monodeoxynucleotide substrates—including dC, dT, dA, dG, dI, and deoxy 5-Nitroindole (dNI)—in combination with both cognate and mismatched bridging templates. Our results reveal a novel activity for T4 DNA ligase (template-directed ligation of a tethered monodeoxynucleotide), and they highlight the suitability of this partition scheme in the selection of ribozymes that phosphorylate mononucleoside substrates.

## Results

### Minimal ligation complexes

To assess templated mononucleotide ligation, minimal substrates were assembled from sets of upstream, downstream and bridging oligonucleotides. The upstream “capture oligo” is an arbitrary 12-nucleotide DNA sequence predicted to contain little or no self-complementarity. The downstream oligonucleotides, referred to collectively as “dXHr8,” carry a single monodeoxynucleoside (dX), such as dC, dT, dA, dG, dI or dNI (e.g., **Fig. 2C** for dCHr8). The dX moiety was tethered through a flexible diphospho-hexaethyleneglycol (HEG) linker to an 8-mer oligoribonucleotide. The exposed 5′ OH group of the tethered dX was readily radiolabeled with ^32^P by polynucleotide kinase (PNK), allowing the substrate to be followed during the course of ligation reactions. Annealing the capture oligo and one of the radiolabeled dXHr8 oligos to any of several bridging oligonucleotides created a minimal double-stranded ligation junction (**Fig. 2B**) that is analogous to a ligation junction that we propose for capturing kinase ribozymes, as in **Fig. 1D**. Several bridging oligos are evaluated in this study, each of which is fully complementary to both the 8-mer RNA segment of dXHr8 and the 12-mer capture oligo. To accommodate the diphospho-HEG spacer within the dXHr8 substrate, these complementary segments are separated by three different kinds of linkers: HEG, a polyA tract or a poly T tract. The bridging templates are accordingly denoted as “HEG-Y,” “A_n_Y” or T_n_Y,” with n denoting the number of unpaired adenosines or thymidines (n = 1, 2, 3, 4, 6, 8, 10) and Y denoting the nucleotide in position to pair with the tethered mononucleotide.

### Optimization of ligation conditions

As a starting point for optimizing the ligations, reactions were assembled containing equal amounts of the seven A_n_G bridge oligonucleotides annealed to the capture oligo and to radiolabeled dCHr8. After overnight incubation with T4 DNA ligase at 10, 20 or 30°C under standard ligation conditions, a small amount (0.4 to 5.6%) of potential full-length ligation product was observed at all three temperatures (**Fig. S1A**). Side products from partial degradation were more prevalent at 30°C, and only a trace amount of ligated product was observed at 10°C. Additional ligations were carried out at 20°C to optimize concentrations of input oligonucleotides and DNA ligase (**Figs. S1B** and **S1C**), and the resulting optimized conditions were used in all subsequent reactions (detailed in Materials and Methods). Each ligation reaction yielded a major product (**Fig. 2D**) that migrated just above a 25 nt DNA marker (compare to later figures). The adenylated mononucleotide intermediate migrated just above the input dCHr8 substrate. An absolute requirement for a 5′-phosphoryl group on the tethered mononucleotide was demonstrated using 3′ radiolabeled dCHr8 with and without prior PNK treatment (data not shown), as expected from the known behavior of DNA ligases.

### Identification of ligation product by RNase T1 analysis

To ensure against misinterpretation of spurious side products that might form with this unnatural ligation substrate—such as cyclization or dimerization of the dXHr8 oligos—we sought to identify which bands correspond to the true ligation products. Although dCHr8 contains a total of 9 nucleotides (8 contiguous nucleotides plus the tethered dC), the added mass of the internal phospho-HEG tether causes dCHr8 to migrate more slowly (**Fig. 2D**, lanes 1 and 9), with an apparent size of approximately 11 nt (not shown). The intended ligation product is susceptible to digestion by RNase T1 on the 3′ sides of the two guanosines in the 8-mer oligo*ribo*nucleotide portion of dCHr8, while the capture oligo is an oligo*deoxyribo*nucleotide and should not be cleaved by RNase T1. Radiolabeled dCHr8 and the putative ligation product were each purified from polyacrylamide denaturing gels. Upon digestion with RNase T1, the radiolabeled product from the dCHr8 digestion migrates just above the dye front, as though with an apparent size of a few (3 to 5) nt (**Fig. 2D**, lanes 2-4), consistent with a product containing radiolabeled dC on one end of the phospho-HEG tether and guanosine on the other end. The lowest concentration of RNase T1 yielded a doublet (**Fig. 2D**, lane 2) resulting from cleavage at position +2 and incomplete digestion following the G at position +1. RNase T1 digestion of the expected DNA/RNA hybrid product is expected to generate a product containing 14 nucleotides (12 deoxynucleotides from the capture oligo plus the radiolabeled [^32^P]-dC and one additional guanosine) in addition to the internal phospho-HEG tether. Indeed, digestion of the putative ligation product yielded a single major band migrating below the undigested ligation product and a few nucleotides above the radiolabeled dCHr8 (**Fig. 2D**, lanes 7 and 8). These results strongly support the identity of the major band as being the ligation product.

### ATP stimulates dCHr8 adenylation but lowers overall ligation rate and yield

**Figure 3:**
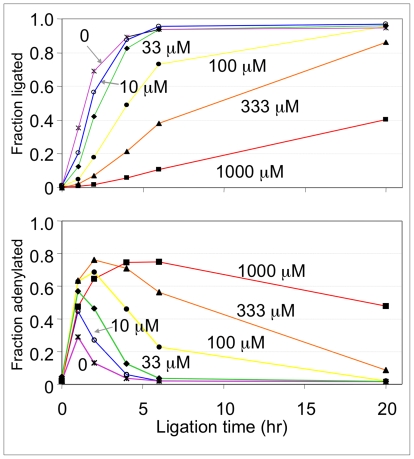
ATP titration. Ligation reactions were carried out with radiolabeled dCHr8 and non-labeled A_2_G bridge in the presence of ATP at concentrations of 0 (asterisks), 10 (open circles), 33 (diamonds), 100 (filled circles), 333 (triangles) or 1000 (squares) µM. **Top**, plot of the fraction of dCHr8 converted to full-length ligation product vs. time at the ATP concentrations indicated next to each curve in micromolar units. Reactions were carried out at 20°C with T4 DNA ligase concentration fixed at 3.7 µM. Samples were taken at 0, 1, 2, 4, 6, and 20 hours. **Bottom**, plot of the accumulation of adenylated intermediate in the same reactions shown in the top panel.

When ATP concentration was varied, both the fraction of substrate converted into ligated product and the rate of this conversion were greatest at the lowest concentrations of ATP (**Fig. 3** **, top**). At 0, 10 or 33 µM ATP, over 80% of the input dCHr8 oligonucleotide is converted to ligated product within 6 hours. With increasing concentration of ATP, a side product consistently accumulated (**Fig. 3** **, bottom**). Assignment of this side product as being the adenylated form of dCHr8 is based on the fact that it migrates approximately one nucleotide above the input substrate, that its formation is sensitive to ATP concentration, and that it chases into ligated product. Note that commercial T4 DNA ligase is purified in a preadenylated form [24]. Thus, while the complete reaction cycle requires ATP, the enzyme (4 µM) is in molar excess over the substrate (3.3 µM), and these reactions proceed under stoichiometric rather than multiple-turnover conditions.

### A two-nucleotide spacer optimally accommodates the phospho-HEG tether

The number of unpaired nucleotides in the bridging oligonucleotide is expected to be a critical determinant of ligation efficiency. A bridging oligonucleotide with too many unpaired nucleotides in a single-stranded stack would place the 3′ OH beyond the reach of the HEG tether, while a bridge with too few nucleotides would invite steric and electrostatic clash between the two phosphates flanking the HEG moiety. To test the influence of spacer length and composition, separate reactions were assembled using 5′ radiolabeled dCHr8 substrate in combination with all sixteen bridge oligonucleotides: HEG-G, A_n_G and T_n_G (n = 0, 1, 2, 3, 4, 6, 8, and 10). Within each series, the fastest rate and highest product yields were obtained with bridges containing two unpaired nucleotides (A_2_G and T_2_G bridges) across from the phospho-HEG tether, with over 80% and 90% conversion to products at 6h, respectively (**Table 1**, **Fig. S2** and data not shown). Yields decreased according to the trends: A_2_>A_1_>(A_3_≈HEG)>A_4_>A_0_>A_6_≫A_8_≈A_10_ and T_2_>T_3_>T_4_>(T_6_≈T_1_)>(HEG≈T_8_)≫T_10_. Unpaired thymidines across from the HEG tether consistently resulted in significantly higher ligation yields than unpaired adenosines at these positions. Placing a HEG linker in the bridge across from the HEG linker in the dXHr8 oligonucleotide gave ligation yields that were comparable to the A_3_G bridge and that were slightly above those of the T_8_G bridge. For bridges T_2_G, T_3_G, A_2_G and (to a lesser extend) T_4_G, reactions were nearing completion at 6 h. However, the other data sets required 20 h or longer to plateau (**Table 1**). Thus, these reactions are considerably slower than reactions involving ligases with nicked DNA substrates, even though both sides of the ligation junctions are Watson-Crick base paired deoxynucleotides (dC/dG).

**Table 1:** Effect of spacer length in the DNA bridge on the ligation efficiency.^a^

	Yield (%)					
	2h		6h		20h	
BRIDGE OLIGO	LIGATION	ADENYLATE	LIGATION	ADENYLATE	LIGATION	ADENYLATE
A_0_G	**0.4**	12	**5.8**	17	**19**	9.9
A_1_G	**15**	36	**69**	12	**87**	3.6
A_2_G	**31**	37	**84**	7.3	**91**	2.9
A_3_G	**11**	14	**39**	7.6	**63**	6.8
A_4_G	**2.8**	13	**20**	11	**48**	4.6
A_6_G	**0.7**	6.7	**1.4**	8.5	**9.1**	10
A_8_G	**0.0**	6.0	**0.0**	6.6	**0.8**	6.8
A_10_G	**0.0**	5.9	**0.0**	6.9	**0.1**	11
T_1_G	**9.9**	66	**51**	41	**87**	6.0
T_2_G	**86**	8.5	**93**	3.7	**94**	3.2
T_3_G	**31**	32	**84**	7.0	**92**	2.6
T_4_G	**20**	48	**79**	14	**92**	2.7
T_6_G	**10**	44	**59**	24	**88**	3.6
T_8_G	**1.6**	21	**8.1**	37	**43**	20
T_10_G	**0.0**	10	**0.6**	19	**6.0**	30
HEG-G	**11**	37	**42**	35	**69**	29

a Reactions using dCHr8 were performed at 20°C using 3.3 µM [^32^P]-labeled dCHr8, 4.1 µM of capture oligo and 4.1 µM of the indicated bridging template. All bridging oligos contained dG in the pairing position across from the tethered dC.

### Specificity of ligation at the minimal junction

**Figure 4:**
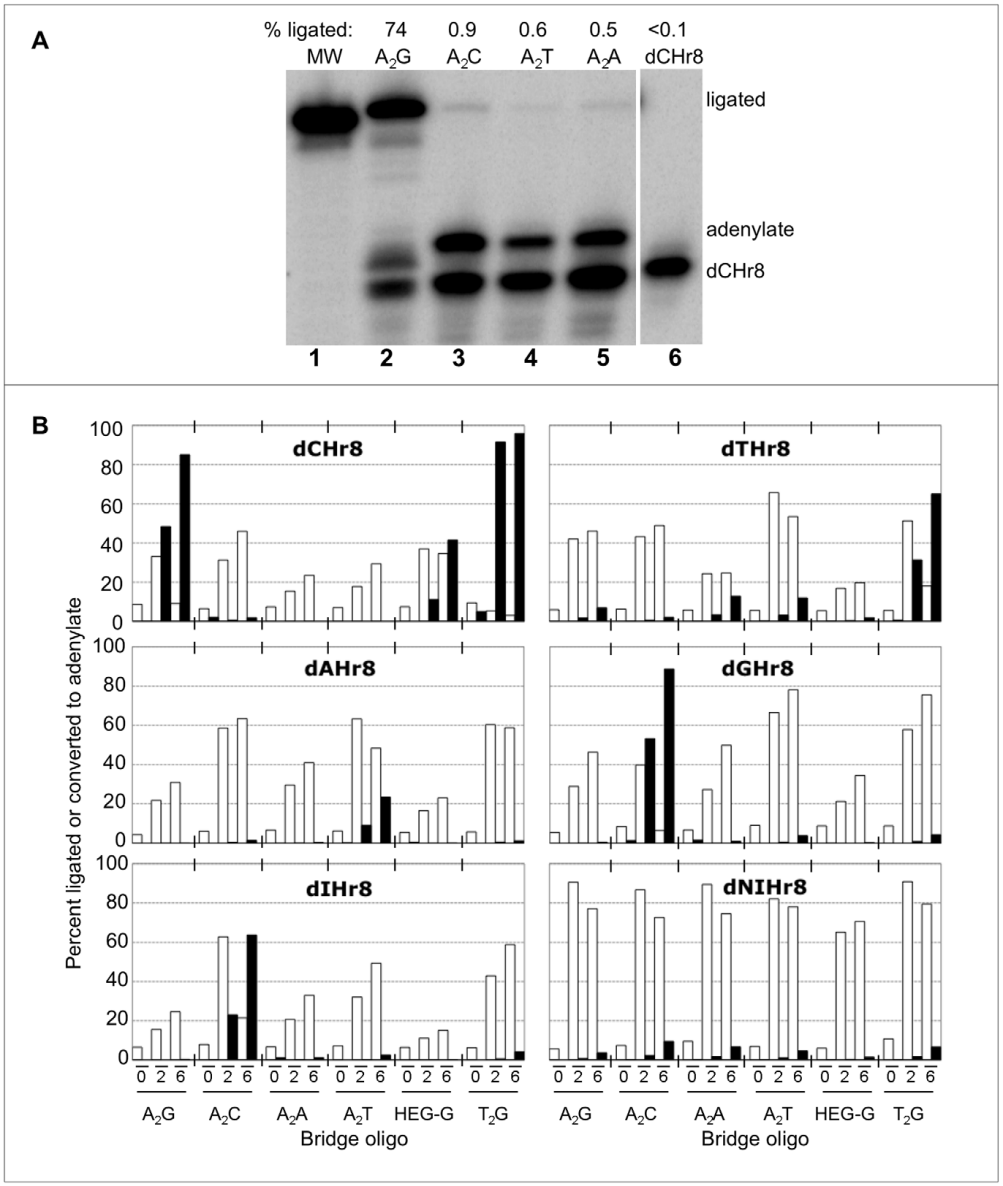
Ligation fidelity. **A**) Representative phosphorimage of ligation reactions using radiolabeled dCHr8 and bridging oligos with each of the four potential templating nucleotide across from the tethered dC. Lane 1, 25 nt DNA size marker. Lanes 2–5, products of 24 h ligation reactions under optimized conditions using the A_2_Y bridge template strand indicated above the lanes. Lane 6, unreacted input dCHr8 substrate. The percent of dCHr8 converted to full-length ligation product for each reaction is shown above the gel. **B**) Yields of adenylates (open bars) and ligated products (filled bars) at 0, 2, and 6 h ligation in the presence of different matching and mismatching A_2_Y bridge templates (indicated below the plots) in separate reactions with each dXHr8 oligo (indicated within each panel).

Ligation fidelity was first examined by comparing yields for radiolabeled dCHr8 annealed to bridging oligonucleotides with matched (A_2_G) or mismatched (A_2_A, A_2_C and A_2_T) junction nucleotides. The matched combination yielded over 70% ligated product, while the mismatched combinations yielded 0.5 to 0.9%, for a discrimination of approximately 100-fold (**Fig. 4A**). This analysis was then expanded to include all combinations of junction nucleotides dX/(A_2_Y), where dX = dC, dT, dA, dG, dI or dNI in the tethered mononucleotide, and Y = dA, dC, dG or dT in the bridging oligonucleotide. The ligation complexes were assembled by annealing the capture oligo with each of the dXHr8 substrates and with all four A_2_Y bridge oligonucleotides in separate reactions. Tethered dNI mononucleotide was included in the set because it is reported to pair promiscuously when used in PCR primers [25], [26], [27], [28]. Two additional bridging oligonucleotides—HEG-G or T_2_G—were included that carried a dG in the pairing position with either HEG or a T_2_ spacer, respectively, across from the HEG tether to determine the effect of spacer composition on the fidelity.

For combinations involving Watson-Crick base pairs at the ligation junction, yields of full-length ligated product ranged from 13 to 89% following the 6 h reactions (**Fig. 4B**). Product yield was greatest for tethered dG and decreased in the order dG>dC>(dI≈dT)>dA≫dNI, with almost no full-length product for the tethered dNI. The identity of the spacer also influenced the ligation. For tethered dC, product yield was greatest for the T_2_G (96% conversion to product in this experiment) and A_2_G bridging oligos (85%), followed by the HEG-G bridge (42%). Reactions involving tethered dT were unusual, in that significantly more ligated product was observed for a T∶G wobble pair than for a Watson-Crick T∶A pair, and while the T_2_G bridge gave significantly more ligated product (65%) than any other combination, yield for the A_2_G bridge was barely above background. All other mismatched pairs had significantly lower yields of ligated product (<3% yield) and greater accumulation of adenylate intermediate in comparison with the matched pairs. Thus, while the tethered, mismatched mononucleotide can participate in the second step of the ligation reaction (substrate adenylation) under appropriate conditions, fidelity appears to be enforced by partial or complete blockage in the third step (nick sealing).

### Kinetic rate constants for Watson-Crick paired substrates

**Figure 5:**
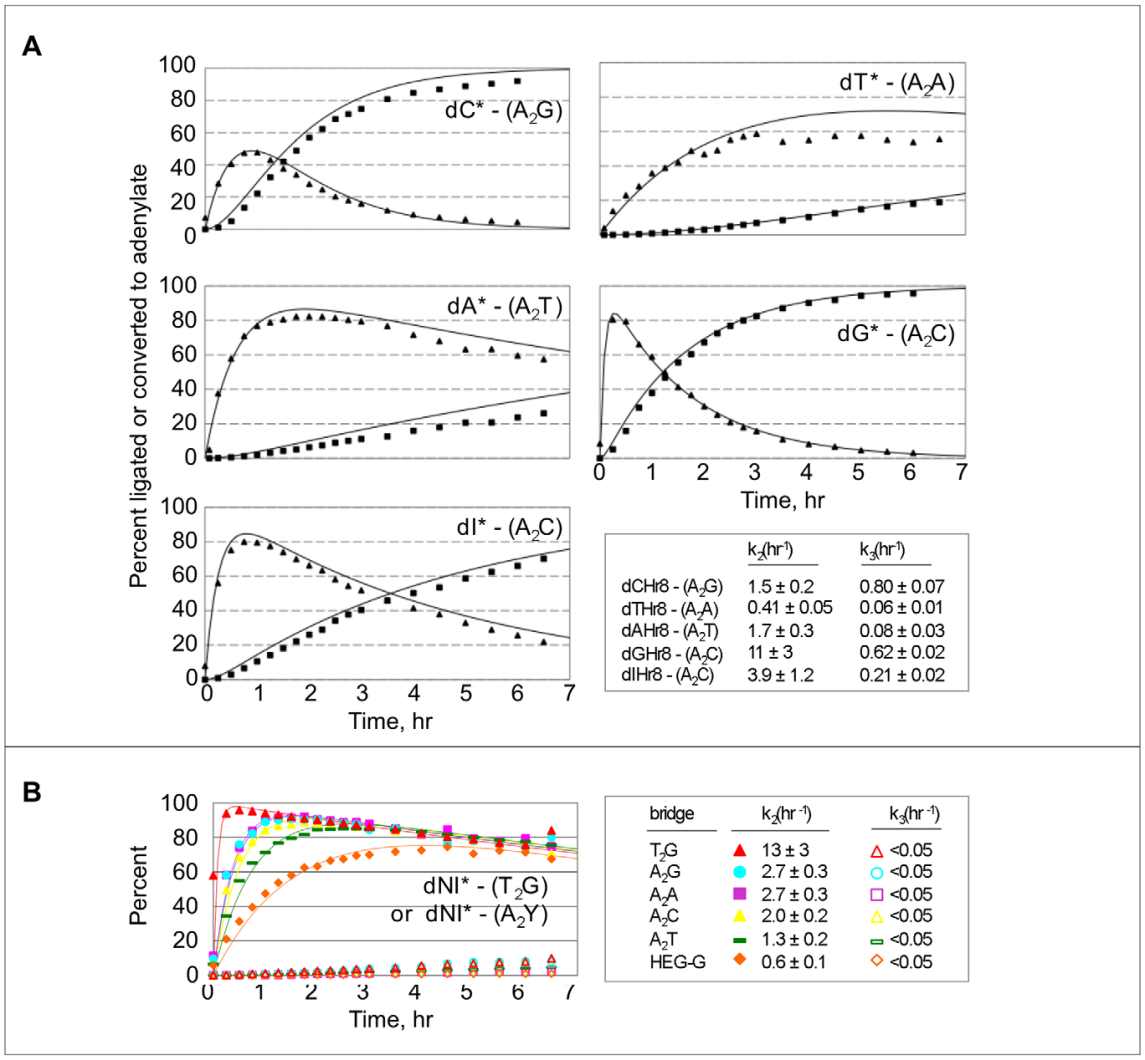
Ligation kinetics. **A**) Ligation reactions were performed at 20°C using 3.3 µM ^32^P-labeled dXHr8, 4.1 µM capture oligo and 4.1 µM of the Watson-Crick-matched DNA bridging template strands. Squares, ligated product; triangles, adenylated intermediate. Identities of the tethered nucleotide (asterisks) and bridges (in parentheses) are indicated. Values of kinetic rate constants k_2_ (5′ DNA adenylation) and k_3_ (gap-sealing) are given in the bottom right panel. **B**) Ligation reactions for tethered “universal” nucleoside, 5-nitroindole (dNI), were performed under the same conditions as in (A) using ^32^P-labeled dNIHr8 in the presence of the indicated bridging template. Filled symbols, adenylate; open symbols, ligated product.

To dissect quantitatively the effects of tethered mononucleotide identity on the rates of adenylate formation and gap-sealing, ligation complexes were assembled with each radiolabeled dXHr8 substrate using the corresponding Watson-Crick paired A_2_ bridges, and samples were collected every 15 to 30 minutes for 6 h. Product yields were again greatest for tethered dG (96%) and dC (92%) and lowest for tethered dA (24%) and dT (18%), while tethered dI gave an intermediate yield (66%). Fitting the data to kinetic equations for a two-step sequential reaction revealed that adenylation (k_2_) is faster than gap-sealing (k_3_) for each of the ligation substrates (**Fig. 5A**). With the exception of dTHr8, all k_2_ values are greater than 1.0 h^−1^. For the gap-sealing step, reactions with tethered dC (0.80 h^−1^) and dG (0.62 h^−1^) were the fastest, and the reactions were the slowest with tethered dA (0.075 h^−1^) and dT (0.06 h^−1^). The value of the gap-sealing rate for tethered dI (0.21 h^−1^) was again intermediate. Reactions involving tethered dNI formed little or no ligated product, irrespective of the bridging oligo used (all k_3_ values<0.05 h^−1^, and all ligation yields <10%), although adenylation was rapid for all bridge combinations (k_2_ ranging from 0.6 h^−1^ to >10 h^−1^) (**Fig. 5B**). Thus, the data for both the Watson-Crick combinations and the tethered dNI establish that the gap-sealing step largely determines the yield of ligated product.

### Multiplexed ligations

In principle, mononucleoside kinase ribozyme discovery could be accelerated though a multiplexed platform in which multiple dXHr8 oligos are attached to the evolving library and all products of RNA-catalyzed phosphoryl transfer are captured in a single ligation. To explore this possibility, oligonucleotide mixtures were evaluated in three formats, designated Mock1, Mock2 and Mock3 (detailed in Materials and Methods). In each format, the six dXHr8 oligos, only one of which was radiolabeled, were annealed with various combinations of A_2_Y bridging oligos. The Mock1 reaction utilized only the bridge that was complementary to the radiolabeled species. Both the bridge and capture oligos were in slight excess of the labeled substrate (oligo ratio of 1∶1.25∶1.25 relative to the labeled dXHr8 substrate). Reactions that included labeled dCHr8 or dGHr8 were the most efficient (>60% yield after 20 h), while the other three were much less efficient (**Table 2**), probably as a result of faster gap-sealing kinetics of these two substrates relative to the others (see above). The Mock2 reactions removed potential competition with the bridging oligo by raising the concentrations of the bridge and capture oligos to be in stoichiometric excess of all dXHr8 present in the reaction (oligo ratio of 1∶1.25∶1.25 relative to ∑(dXHr8)_i_). Under these conditions, nearly all of labeled dGHr8 and >50% of labeled dIHr8 became ligated, but only about one-third of labeled dCHr8 became ligated and less still for tethered dA and dT substrates (**Table 3**). To determine whether all phosphorylated products could be captured in a single ligation, a Mock3 series was carried out in which each reaction included a mixture of all four A_2_Y Bridge oligonucleotides (A_2_G, A_2_C, A_2_T, and A_2_A), and five of the tethered oligo substrates (dCHr8, dGHr8, dIHr8, dAHr8 and dTHr8—only one of which at a time was radiolabeled) at a ratio of 1∶1.25∶1.25. As observed above for the Mock1 reactions, the greatest conversion to ligated product was observed for labeled substrates carrying tethered dC or dG. Reactions wherein the dT oligo was labeled were substantially improved relative to Mock1 or Mock2 conditions, while those carrying labeled dA or dI were equivalent or poorer in comparison to Mock1 or Mock2 conditions (**Table 4**). Lack of strong inhibition by the misannealed substrates may indicate that the ligation complexes are in dynamic exchange, and that proper annealing strongly favors product formation. In sum, each of the three simulated multiplexed reactions allows the recovery of more than one phosphorylated product and could, in principle, be used to recover multiple families of kinase ribozymes from a single multiplexed selection. However, even under the best conditions, the 4-fold difference between the most (dC or dG) and the least (dT) efficiently ligated product could bias the selection outcome.

**Table 2:** Simulated Multiplex Ligations: Mock1.^a^

	2 h		4h		6h		8h		20h	
	LIG	ADEN	LIG	ADEN	LIG	ADEN	LIG	ADEN	LIG	ADEN
^32^P-dCHr8	**5**	36	**14**	41	**26**	35	**40**	31	**76**	9
^32^P-dTHr8	**0.03**	13	**0.16**	17	**0.37**	21	**1**	26	**6**	36
^32^P-dAHr8	**0.07**	22	**0.45**	35	**1**	45	**2**	52	**14**	53
^32^P-dGHr8	**5**	74	**12**	77	**18**	69	**28**	60	**61**	23
^32^P-dIHr8	**0.36**	35	**2**	49	**4**	57	**7**	60	**26**	46

a Capture oligo and complementary bridging template were in slight excess of the radiolabeled strand. The species that carried the radiolabel is indicated in the first column.

**Table 3:** Simulated Multiplex Ligations: Mock2.^a^

	2 h		4h		6h		8h		20h	
	LIG	ADEN	LIG	ADEN	LIG	ADEN	LIG	ADEN	LIG	ADEN
^32^P-dCHr8	**9**	19	**16**	13	**22**	10	**28**	9	**35**	6
^32^P-dTHr8	**0.25**	13	**1**	16	**2**	16	**2**	18	**7**	15
^32^P-dAHr8	**1**	34	**3**	41	**6**	41	**10**	38	**20**	27
^32^P-dGHr8	**32**	53	**57**	31	**71**	18	**80**	10	**90**	4
^32^P-dIHr8	**5**	50	**12**	51	**20**	46	**29**	38	**55**	14

a Capture oligo and complementary bridging oligo were in slight excess of all dXHr8 species present in the reaction.

The species that carried the radiolabel is indicated in the first column.

**Table 4:** Simulated Multiplex Ligations: Mock3.^a^

	2 h		4h		6h		8h		20h	
	LIG	ADEN	LIG	ADEN	LIG	ADEN	LIG	ADEN	LIG	ADEN
^32^P-dCHr8	**15**	19	**35**	18	**52**	14	**63**	10	**83**	5
^32^P-dTHr8	**0.3**	31	**1**	43	**3**	53	**4**	60	**21**	59
^32^P-dAHr8	**0.6**	38	**2**	55	**4**	60	**6**	64	**22**	59
^32^P-dGHr8	**2**	68	**6**	79	**14**	75	**27**	63	**61**	31
^32^P-dIHr8	**1**	30	**2**	47	**5**	68	**10**	68	**29**	48

a All five dXHr8 species were present in each reaction. The species that carried the radiolabel is indicated in the first column. All bridging oligos were also present (A_2_C, A_2_G, A_2_T, and A_2_A). Each matching bridge was in slight excess of the corresponding dXHr8 species.

## Discussion

The present study demonstrates that bacteriophage T4 DNA ligase can join tethered monodeoxynucleotides to the 3′ end of a “capture” oligo in a template-directed fashion, in analogy with a proposed partition step for in vitro selection of kinase ribozymes that phosphorylate tethered monodeoxynucleosides. Optimal joining at 20°C was achieved in low ATP concentrations (≤33 µM), high ligase (0.67 U/µL) concentrations, and at a ratio of 1∶1.25∶1.25 for the three nucleic acid strands (dXHr8: capture oligo: bridging strand). DNA ligases often act as repair enzymes to seal nicks in long duplex DNA. The tethered mononucleotide junctions are sub-optimal substrates for the enzyme; thus, the observed requirements can be understood in terms of preventing re-adenylation of enzyme that dissociates from an adenylated intermediate before sealing the nick. Although the overall ligase reaction cycle requires ATP, the enzyme is purified in adenylated form and does not require recharging for the single-turnover reactions (enzyme in excess) described here and in several of these prior works. The relative rates of gap-sealing, enzyme dissociation and re-adenylation control product distribution for other suboptimal ligation junctions, such as Chlorella virus DNA ligase at gapped [29] or nicked [30] substrates, T4 DNA ligase at mismatched or blunt-ended junctions or in the absence of an upstream fragment [31], [32], [33], RNA-templated DNA ligations [34], and mammalian DNA ligase I with a 5′-p(rA) ribonucleotide on the downstream fragment [35].

**Figure 6:**
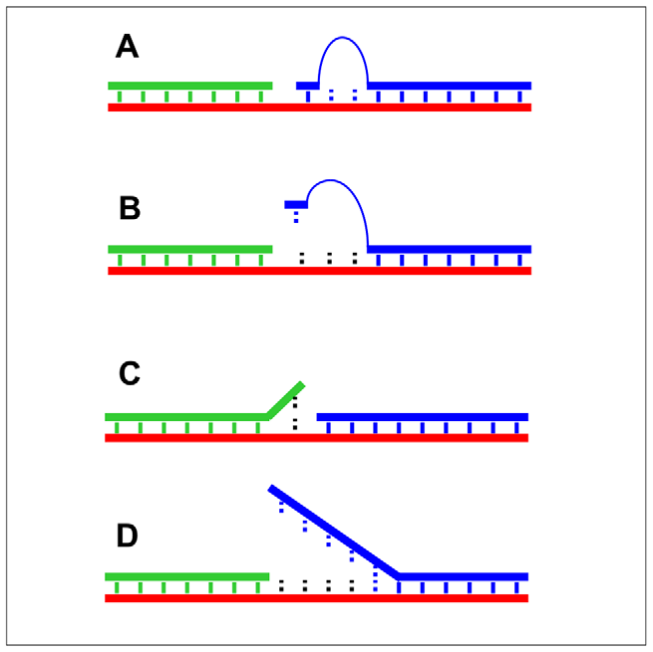
Matched and mismatched ligation junctions. **A**) Minimal ligation junction with Watson-Crick paired tethered mononucleotide using the A_2_Y or T_2_Y bridge. **B**) Single nucleotide mismatch in the minimal ligation junction such as those in lanes 3–5 of Figure 4A, **C**) Single nucleotide mismatch on the upstream side of the ligation junction (not efficiently ligated by T4 DNA ligase), and **D**) Up to 5 nt DNA mismatches on the downstream side of the ligation junction (ligated at moderate efficiency by T4 DNA ligase at low ATP concentrations).

The identity of the tethered mononucleotide and its pairing partner in the bridging oligo strongly affected kinetic rate constants for adenylate formation (k_2_) and gap-sealing (k_3_), and thereby determined the overall efficiency and fidelity of the ligation reaction. Gap-sealing was fastest for tethered dC and dG, followed by tethered dI, and it was considerably slower for tethered dT and dA. Similar trends in product yields were evident in the multiplexed reactions and can be understood in terms of the relative kinetic rate constants for the individual reactions. For tethered dT, a higher ligation yield was obtained using the T_2_G bridge than any of the other bridges. Ligations involving tethered mononucleotides exhibited approximately 100-fold preference for most Watson-Crick paired substrates over mispaired substrates (**Fig. 6A and 6B**). These differences may be understood in terms of helical geometry within the active site of the enzyme. For example, mismatched pairs often protrude into the minor groove, and fidelity of the *Tth* ligase from *Thermus thermophilus* HB8 is enforced by interactions of the protein with the minor groove of the DNA duplex [23]. The “universal” base dNI, which has found utility within PCR primers [25], [26], [27], [28], was efficiently adenylated but did not support gap-sealing under these conditions, perhaps due to distortions of local helical structure.

T4 DNA ligase is especially sensitive to mismatches on the upstream side of the ligation junction (**Fig. 6C**), and this sensitivity provides the basis for diagnostic tests for single nucleotide polymorphisms [31], [36], [37]. The human DNA ligase IV/XRCC4 complex shows a similar sensitivity to upstream mismatches [38]. Sensitivity to covalent nucleotide modifications has also been developed into an assay for detecting modified cellular RNA [39]. In contrast, mismatches on the downstream side of a ligation junction in duplex DNA (**Fig. 6D**) are more readily tolerated [31], [34]. In a previous study, nearly all possible combinations of downstream single-nucleotide mismatches yielded >80% ligated product under conditions of low ATP concentrations even with multiple consecutive mismatches [22], [31]. This tolerance for mismatches on the downstream side is sharply contrasted by the results obtained in the present study, where a single tethered nucleotide provides the interaction energy on the downstream side. The tethered mononucleotide ligation reaction therefore displays greater overall fidelity than reactions with conventional mismatched nicked-DNA substrates [22], [31], [36].

**Figure 7:**
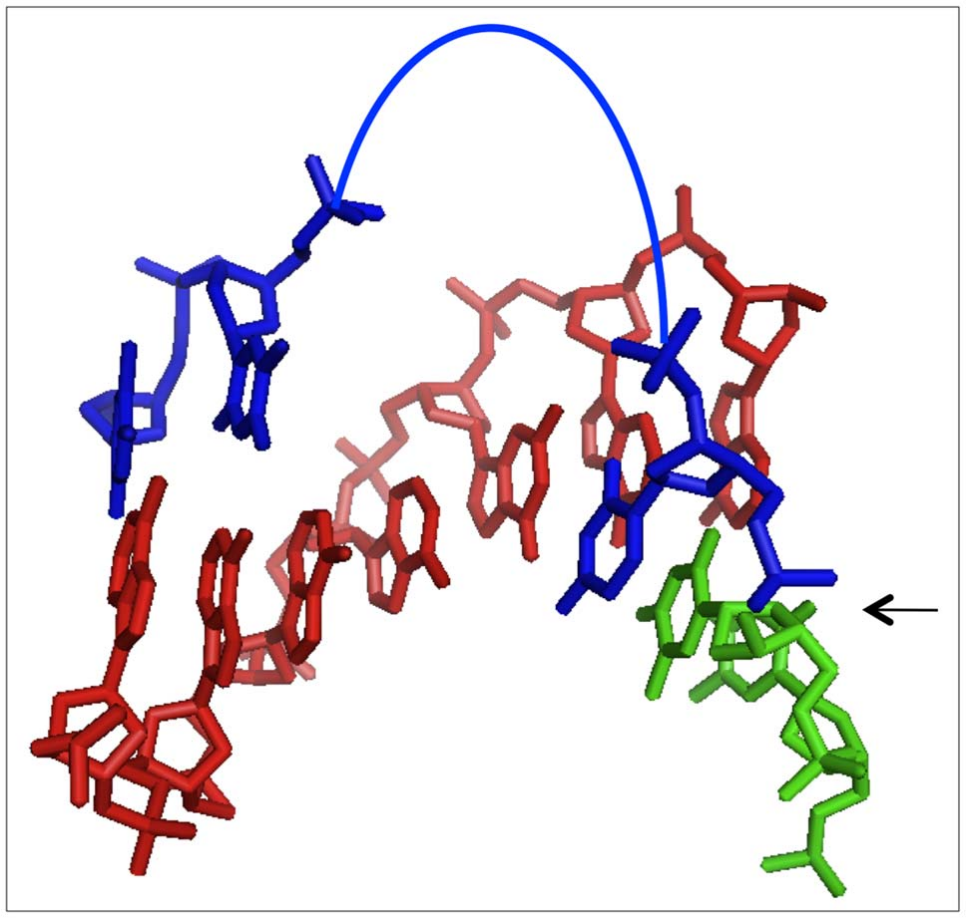
Model of 1nt ligation junctions. Structural model of tethered mononucleotide ligation junction, color coded as in Figures 1 and 6. Arc, HEG linker; arrow, ligation junction. Non-templating spacer nucleotides are shown as standard B-form DNA. Helical axis is horizontal; flanking nucleotides are omitted for clarity.

Ligation of the tethered mononucleotides was optimal when the bridge oligo contained two adenosines or thymidines across from the HEG tether. The calculated P-to-P distance for two base pairs along a B-form helix is very similar to the calculated “average” P-to-P distance in the HEG (∼19Å) if the tether is assumed to be fully flexible (see Materials and Methods), suggesting that the trajectory of the non-templating spacer nucleotides in the bridging strand approximates a normal B-form helix, as in a single-strand stack (**Fig. 7**). A HEG spacer in the bridge (“HEG-G”) was slightly less favorable, giving product yields comparable to an A_3_G bridge. Product yields were invariably higher when unpaired thymidines, rather than adenosines were in the bridge across from the HEG-tether. For bridge oligonucleotides with large spacers (e.g. A_6_ or T_6_ and larger), the polyA or poly T regions are too large to continue a B-form trajectory. The low yield for the larger A_n_X bridges suggest that the poly A tracts resist looping out, instead forming a single-stranded stack of adenosines. In contrast, the poly T tracts appear to be more flexible, as some ligation product is detected even with T_10_G bridge oligonucleotide at long incubation times.

The high yields observed here and T4 DNA ligase's strict requirement for a 5′ phosphate satisfy the necessary criteria for a ligation-based selection of kinase ribozymes that phosphorylate a tethered DNA nucleoside. It should be possible to generalize this strategy to capture phosphorylated RNA nucleosides and even prodrug analog nucleosides. Knowledge of relative kinetics will aid appropriate selection design to avoid excessive recovery biases. We therefore anticipate that further improvements in library design and selection methodology will yield ribozymes for nucleoside phosphorylation and for other metabolic reactions of interest to synthetic biology.

## Materials and Methods

### Oligonucleotides, enzymes and reagents

DNA oligos were purchased from Integrated DNA Technologies (Coralville, IA). The dXHr8 RNA oligonucleotides were purchased from Integrated DNA Technologies, except for dCHr8, which was purchased from Dharmacon, Inc. (Lafayette, CO). Matrix-assisted laser desorption/ionization-time of flight (MALDI-TOF) mass spectrometry analysis of dCHr8, performed at the mass spectrometry core facilities in the Department of Chemistry, Indiana University, identified a single product at the expected m/z ratio of 3149. T4 DNA ligase and T4 polynucleotide kinase were purchased from Epicentre (Madison, WI) and RNasin from Takara International (Madison, WI).

### Polynucleotide kinase labeling

dXHr8 oligos were radiolabeled on the tethered mononucloside with 0.05 to 0.10 U/µL T4 PNK in 1× PNK buffer (50 mM Tris pH 7.6, 66.7 mM KCl, 10 mM MgCl_2_, 1 mM dithiothreitol) at 37°C with 2.4 µM [γ-^32^P] ATP (3000 or 7000 Ci/mmol). After 15 min, non-radioactive ATP was added to 2.5 mM final concentration and reactions were continued for an additional 15 minutes to ensure complete 5′-phosphorylation. Labeled products were purified from denaturing (8M urea) polyacrylamide gels (12–15%), precipitated and resuspended in water. Specific activities were adjusted to approximately 1–2×10^5^ cpm/pmol with non-radioactive, 5′-phosphorylated oligonucleotides.

### Optimization of ligation conditions

Reactions were optimized progressively with respect to temperature and to the concentrations of ligase, ATP and oligonucleotide substrates. *Temperature*: Initial pilot reactions contained 17.5 µM radiolabeled dCHr8, 17.5 µM unlabeled capture oligo and 2.5 µM of each of seven bridging oligos (A_0_G, A_1_G, A_2_G, A_4_G, A_6_G, A_8_G, A_10_G). Oligo mixtures were denatured in water at 75°C for 3 min, then cooled on ice to anneal ligation complexes. Buffer was added to yield final concentrations of 1 mM ATP, 0.02 U/µL RNasin, 50 mM Tris pH 7.6, 66.7 mM KCl, 10 mM MgCl_2_ and 1 mM dithiothreitol. Ligation was initiated by addition of T4 DNA ligase to a final concentration of 6 µM ( = 0.33 µg/µL = 1U/µL) on ice, followed by overnight incubation at 10, 20 or 30°C. Reactions were stopped by adding an equal volume of gel-loading buffer (92% formamide, 20 mM EDTA, 0.01% bromophenol blue and 0.01% Xylene cyanol). Samples were separated on 10% denaturing polyacrylamide gels, which were dried and exposed to phosphorimager plates. Gel data were analyzed with ImageQuant software (Molecular Dynamics) by dividing the signal in each band (ligated product, adenylate, unreacted) by the total signal for all bands within the lane. Temperature was held at 20°C for all subsequent reactions. *Ligation complex concentration*: dCHr8, capture oligo and the A_2_G bridging oligo were mixed at ratio of 1∶1.25∶1.25, and assayed as above for ligation. Ligated product improved markedly at dCHr8 concentrations between 0.1 µM and 1.0 µM, with little further increase between 1.0 µM and 10 µM (**Fig. S1B**). Concentration of dXHr8 was held constant at 3.3 µM for subsequent reactions. *Ligase concentration*: Reactions were assembled for dCHr8/capture oligo/A_2_G bridge and incubated overnight (20 h) in various concentrations of T4 DNA ligase. The fraction ligated increased as enzyme concentration was increased from 0.06 to 6 µM (**Fig. S1C**). Yield approached saturation at 4 µM, which is the lowest concentration at which enzyme was in stoichiometric excess of dCHr8 (see Discussion). This concentration was used in subsequent reactions. *ATP concentration*: Finally, reactions were assembled for dCHr8/capture oligo/A_2_G bridge with added ATP concentrations ranging from 0 µM to 1000 µM, as detailed above (**Fig. 3**). For reactions carried out at 20°C, optimal joining was achieved with two non-annealed nucleotides separating the tethered mononucleotide from the annealed 8-mer, at low ATP concentrations (≤33 µM, to avoid enzyme re-adenylation) and high ligase concentrations (0.67 U/µL, in molar excess of annealed complexes), and at a ratio of 1∶1.25∶1.25 for the three nucleic acid strands (dXHr8: capture oligo: bridging strand).

### Identification of ligation product by RNase T1 analysis

Samples of dCHr8 or full-length ligation products were diluted to 1.25 µM into aliquots of 1× T1 RNase buffer (20 mM sodium citrate, 1 mM EDTA, 7M urea, 180 µg/mL tRNA) that contained 0, 0.01, 0.1 or 1.0 U/µL RNase T1 (Boehringer Mannheim). Samples were incubated at 37°C for 30 minutes and analyzed by denaturing gel electrophoresis and phosphorimaging as described above.

### Rate and kinetics of ligation of different mononucleotides

To derive the rate constants for the adenylation and gap-sealing reactions, aliquots were withdrawn from ligation reactions every 15 min until 3 h and every 30 min between 3 h and 6 h. The fraction converted to adenylate and to ligated product were fit to standard kinetic equations for consecutive reactions of the form A→B→C by manually adjusting the values of k_2_ (A→B, adenylate formation) and k_3_ (B→C, gap-sealing reaction):
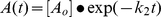
(1)

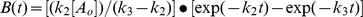
(2)

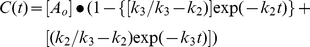
(3)Enzyme charging was not considered, since pre-charged ligase was used in stoichiometric excess in single-turnover reactions. Low-level degradation of input substrate and the ligated product led to undersampling at long time points in some reactions, making the curve-fitting most reliable at early times.

### Multiplexed ligations

For the Mock1 experiments, one radiolabeled and five non-labeled dXHr8 oligos were included at 3.3 µM each (19.8 µM total final concentration); capture and complementary bridge oligos were included at 4.1 µM each. For the Mock2 experiments, capture and bridging oligos were each raised to 24.6 µM to ensure annealing to all six dXHr8 species. For the Mock3 experiments, one radiolabeled and five non-labeled dXHr8 oligos were included at a final concentration of 0.55 µM each (total 3.3 µM) and capture oligo at 4.1 µM, in addition to one matched and three mismatched A_2_Y bridges (A_2_C, A_2_G, A_2_T and A_2_A), each at a final concentration of 1.025 µM (4.1 µM total) thus maintaining the net oligo ratio of 1∶1.25∶1.25 relative to total dXHr8. Concentrations of enzyme and other components were held constant for all three formats.

### Conformational modeling

HEG tether was modeled using the spatial reference frame of the P atom on G_+1_ (**Fig. S3A**). The set of all points that can be occupied by the P atom at the other end of the HEG linker describes a sphere (“S”) of radius “R,” where R is the P-to-P distance for the fully extended HEG (approximately 24.5Å). Assuming the chain to be sufficiently flexible that every volume element within the sphere is equally likely to be occupied, the average inter-phosphate distance is defined by a smaller sphere (“s”) whose volume is half that of sphere “S.” The radius of this inner sphere is thus 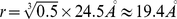. For a B-form DNA duplex, the through-space, P-to-P distances (d) between two phosphates can be calculated from a simple geometric depiction of the cylindrical spiral (**Fig. S3B**) using the relation 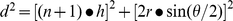; where n is the number of intervening base pairs (irrespective of sequence), h is the rise per base pair (3.3Å for B-form DNA), r is the helical radius (10Å for B-form DNA), and θ is the net rotation around the helical axis (34.6° per base pair rise). For phosphates separated by 0, 1, 2, or 3 intervening base pairs, inter-phosphate distance is calculated to be 6.8, 13.1, 18.6 and 22.8 Å, respectively.

## Supporting Information

Figure S1 Optimization of ligation conditions. A) Temperature effects. Products of ligation using mixed A_n_G bridges/capture oligo/dCHr8. Reactions were incubated overnight at 10°, 20°, or 30°C, as indicated above the lane. B) Effect of substrate concentration. Labeled dCHr8 and unlabeled capture and bridging oligos were used in 1∶1.25∶1.25 ratio, with total dCHr8 concentration ranging from 0.1 to 10 µM, as indicated above the sets of lanes. Samples were taken at 0, 1, 2, 4, 6 and 20 hours. Size markers are radiolabeled 29nt DNA (left) and dCHr8 oligo, (right). C) Ligase titration. T4 DNA ligase was used at various input concentrations (indicated above the lane) in reactions with 3.3 µM dCHr8 and other oligos at 1∶1.25∶1.25 ratio. An enzyme concentration of 0.67 U/µL corresponds to approximately 4 µM. Samples were taken at 0, 1, 2, 4, 6 and 20 hrs.
(6.32 MB TIF)

Figure S2 Evaluation of spacer length in A_n_G bridging oligos. Example of an early evaluation of the effect of spacer length. Each ligation used radiolabeled dCHr8 and the bridging oligo indicated above the lanes. Reactions proceeded for 2 or 20 hours.
(0.52 MB TIF)

Figure S3 Modeling of 1nt ligation junctions. A. Schematic of B-form DNA helix modeled as a cylindrical spiral. Spheres, backbone phosphorous atoms; θ, net rotation about the helix; h, net rise; r, radius. Inter-phosphate distance (yellow line) is given by d∧2 = [(n+1)h]∧2 + [2rsin(θ/2)]∧2, as detailed in Materials and Methods. B. Schematic of HEG linker, modeled as fully flexible chain. Purple spheres, phosphorous atoms at each end of the HEG unit (blue squiggle); R, radius of maximal sphere that could be occupied by fully-extended HEG; r, radius of sphere with HEG unit extended to an average distance. Relationship between the two spheres is (r/R)∧3 = 0.5, as detailed in Materials and Methods.
(0.40 MB TIF)
