# Supplementary figures and images for: Template-Directed Ligation of Tethered Mononucleotides by T4 DNA Ligase for Kinase Ribozyme Selection
Source: PLoS One. 2010 Aug 24;5(8):e12368. doi: 10.1371/journal.pone.0012368 (PMC2927549; doi:10.1371/journal.pone.0012368)

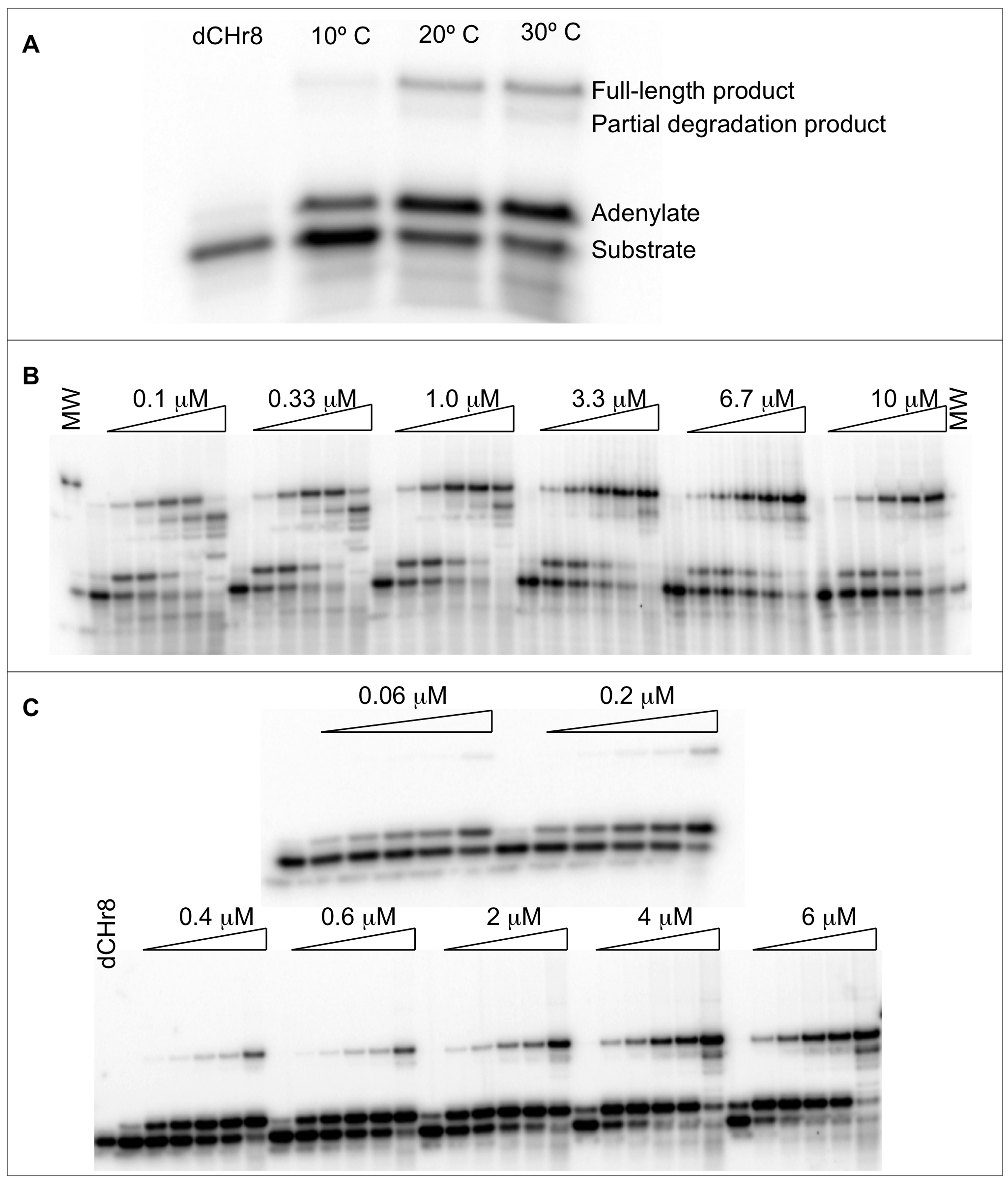

Supplement: Figure S1 — Optimization of ligation conditions. A) Temperature effects. Products of ligation using mixed AnG bridges/capture oligo/dCHr8. Reactions were incubated overnight at 10°, 20°, or 30°C, as indicated above the lane. B) Effect of substrate concentration. Labeled dCHr8 and unlabeled capture and bridging oligos were used in 1∶1.25∶1.25 ratio, with total dCHr8 concentration ranging from 0.1 to 10 µM, as indicated above the sets of lanes. Samples were taken at 0, 1, 2, 4, 6 and 20 hours. Size markers are radiolabeled 29nt DNA (left) and dCHr8 oligo, (right). C) Ligase titration. T4 DNA ligase was used at various input concentrations (indicated above the lane) in reactions with 3.3 µM dCHr8 and other oligos at 1∶1.25∶1.25 ratio. An enzyme concentration of 0.67 U/µL corresponds to approximately 4 µM. Samples were taken at 0, 1, 2, 4, 6 and 20 hrs. (6.32 MB TIF) [file pone.0012368.s001.tif]

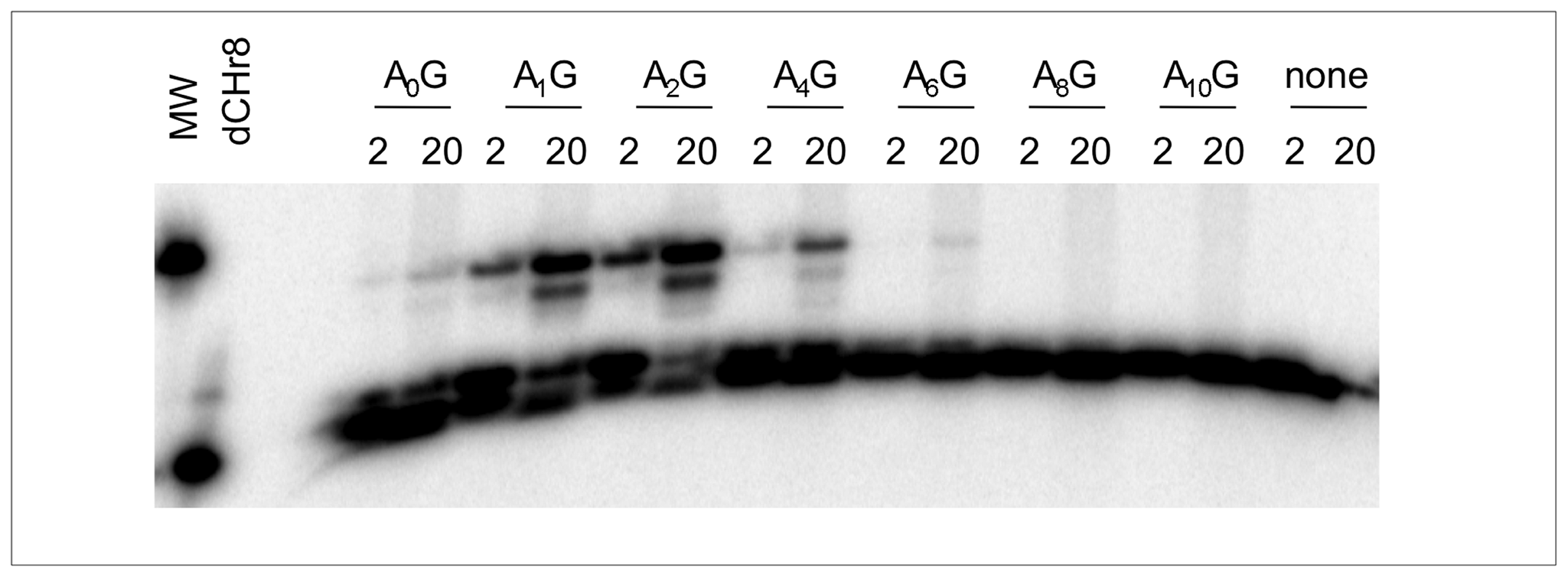

Supplement: Figure S2 — Evaluation of spacer length in AnG bridging oligos. Example of an early evaluation of the effect of spacer length. Each ligation used radiolabeled dCHr8 and the bridging oligo indicated above the lanes. Reactions proceeded for 2 or 20 hours. (0.52 MB TIF) [file pone.0012368.s002.tif]

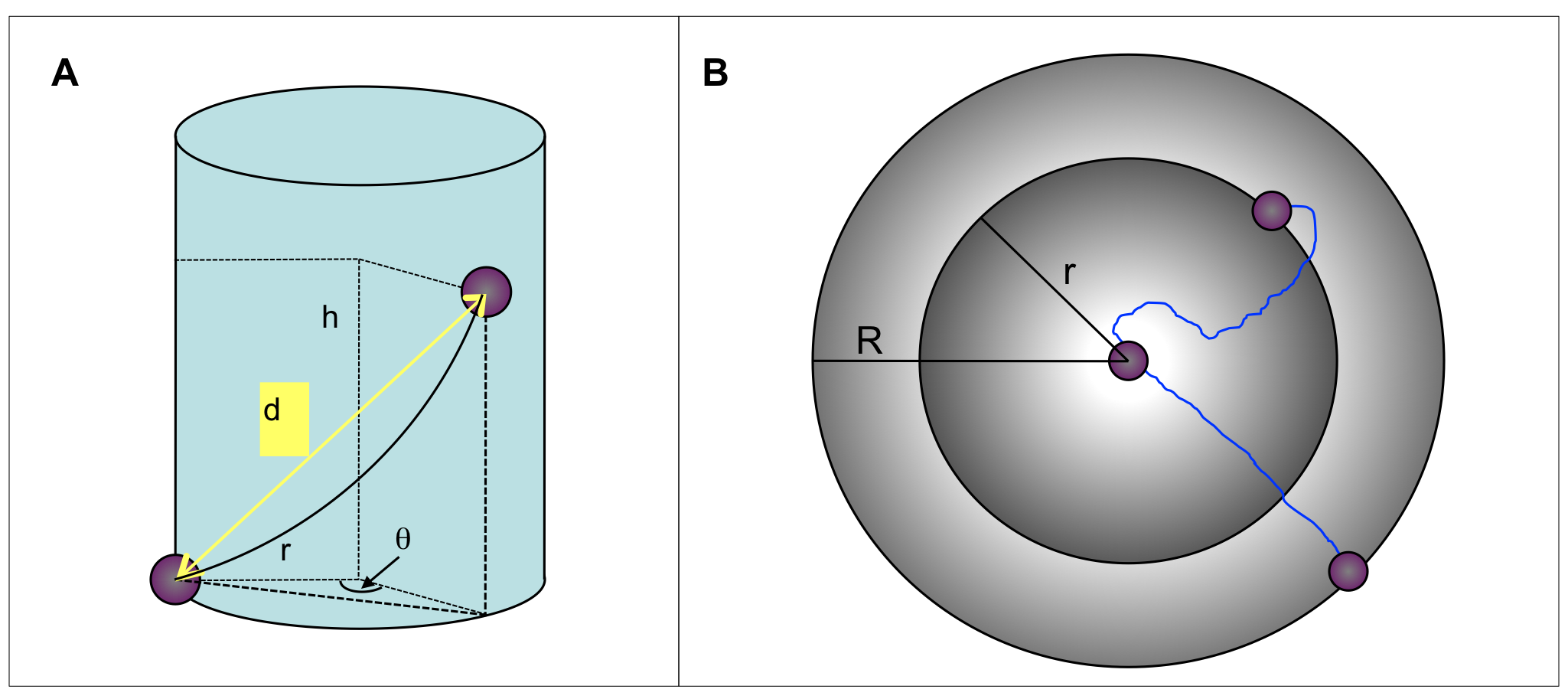

Supplement: Figure S3 — Modeling of 1nt ligation junctions. A. Schematic of B-form DNA helix modeled as a cylindrical spiral. Spheres, backbone phosphorous atoms; θ, net rotation about the helix; h, net rise; r, radius. Inter-phosphate distance (yellow line) is given by d∧2 = [(n+1)h]∧2 + [2rsin(θ/2)]∧2, as detailed in Materials and Methods. B. Schematic of HEG linker, modeled as fully flexible chain. Purple spheres, phosphorous atoms at each end of the HEG unit (blue squiggle); R, radius of maximal sphere that could be occupied by fully-extended HEG; r, radius of sphere with HEG unit extended to an average distance. Relationship between the two spheres is (r/R)∧3 = 0.5, as detailed in Materials and Methods. (0.40 MB TIF) [file pone.0012368.s003.tif]
